# Variable piperaquine exposure significantly impacts protective efficacy of monthly dihydroartemisinin-piperaquine for the prevention of malaria in Ugandan children

**DOI:** 10.1186/s12936-015-0908-8

**Published:** 2015-09-24

**Authors:** Kerstin Sundell, Prasanna Jagannathan, Liusheng Huang, Victor Bigira, James Kapisi, Mary M. Kakuru, Rada Savic, Moses R. Kamya, Grant Dorsey, Francesca Aweeka

**Affiliations:** Department of Pharmaceutical Biosciences, Faculty of Pharmacy, Uppsala University, Uppsala, Sweden; Department of Medicine, San Francisco General Hospital, University of California, San Francisco, USA; Department of Clinical Pharmacy, University of California, San Francisco, USA; Infectious Diseases Research Collaboration, Kampala, Uganda; Department of Bioengineering and Therapeutics, University of California, San Francisco, USA; Department of Medicine, Makerere University College of Health Sciences, Kampala, Uganda

**Keywords:** Malaria, *Plasmodium falciparum*, Chemoprevention, Dihydroartemisinin-piperaquine, Pharmacokinetics

## Abstract

**Background:**

Anti-malarial chemoprevention with dihydroartemisinin-piperaquine (DHA/PQ) is a promising tool for malaria control, but its efficacy in children may be limited by inadequate drug exposure.

**Methods:**

Children were enrolled in a non directly-observed trial of DHA/PQ chemoprevention in a high transmission setting in Uganda. Children were randomized at 6 months of age to no chemoprevention (n = 89) or monthly DHA/PQ (n = 87) and followed through 24 months of age, with pharmacokinetic sampling performed at variable times following monthly dosing of DHA/PQ. A previously published pharmacokinetic model was used to estimate piperaquine (PQ) exposure in each child, and associations between PQ exposure and the protective efficacy (PE) of DHA/PQ were explored.

**Results:**

The incidence of malaria was 6.83 and 3.09 episodes per person year at risk in the no chemoprevention and DHA/PQ arms, respectively (PE 54 %, 95 % CI 39–66 %, P < 0.001). Among children randomized to DHA/PQ, 493 pharmacokinetic samples were collected. Despite nearly 100 % reported adherence to study drug administration at home, there was wide variability in PQ exposure, and children were stratified into three groups based on average PQ exposure during the intervention that was determined by model generated percentiles (low, n = 40; medium, n = 37, and high, n = 10). Gender and socioeconomic factors were not significantly associated with PQ exposure. In multivariate models, the PE of DHA/PQ was 31 % in the low PQ exposure group (95 % CI 6–49 %, P = 0.02), 67 % in the medium PQ exposure group (95 % CI 54–76 %, P < 0.001), and 97 % in the high PQ exposure group (95 % CI 89–99 %, P < 0.001).

**Conclusions:**

The protective efficacy of DHA/PQ chemoprevention in young children was strongly associated with higher drug exposure; in children with the highest PQ exposure, monthly DHA/PQ chemoprevention was nearly 100 % protective against malaria. Strategies to ensure good adherence to monthly dosing and optimize drug exposure are critical to maximize the efficacy of this promising malaria control strategy.

Trial Registration: Current Controlled Trials Identifier NCT00948896

**Electronic supplementary material:**

The online version of this article (doi:10.1186/s12936-015-0908-8) contains supplementary material, which is available to authorized users.

## Background

Despite widespread use of insecticide-treated bednets and case management using artemisinin-based combination therapy (ACT), the incidence of malaria among children continues to be very high in many parts of Africa [1, 2]. With no vaccine available, malaria control centers on vector management and effective use of anti-malarial drugs [3]. Insecticide-treated bednets (ITNs) and indoor residual spraying (IRS) of insecticides are important, but these offer only partial protection, especially in highly endemic areas, in part due to worsening insecticide resistance [4]. The use of effective drugs to treat malaria is critical to control, but in many areas the malaria burden has not improved in recent years [1, 2].

ACT is now the standard therapy for uncomplicated falciparum malaria in nearly all areas, due to widespread resistance to chloroquine and sulfadoxine-pyrimethamine (SP) [5]. ACT combines a potent, but short-acting artemisinin with a longer acting partner drug. Standard ACT across Africa is artemether/lumefantrine (AM/LM) and artesunate/amodiaquine (AS/AQ), and an alternative is dihydroartemisinin/piperaquine (DHA/PQ) [6]. An important role for anti-malarials in addition to treatment of acute illness is intermittent preventative therapy (IPT), the provision of full treatment courses at regular intervals to high risk populations. In Africa, IPT is now standard practice during pregnancy, with doses of sulfadoxine-pyrimethamine (SP) each trimester in endemic areas, although this strategy is seriously limited by resistance to SP [7]. IPT is also recommended in children in some settings (with SP or SP + AQ), but only when resistance to SP is uncommon, which limits this practice principally to the Sahel subregion [8, 9].

Given widespread resistance to SP in parts of Africa, many are considering the use of ACT for IPT, and a logical choice is DHA/PQ, which provides rapid killing of most parasites by DHA and protection for weeks after therapy due to the long half-life of PQ [10]. Directly observed monthly DHA/PQ offered protective efficacy of 98 % against malaria in Thai adults [11]. Likewise, in Uganda, directly observed monthly DHA/PQ administered to school children 6–14 years of age for 1 year had a protective efficacy of 96 % [12]. In contrast, monthly DHA/PQ administered without directly observed therapy to children 6–24 months of age had a protective efficacy against malaria of 58 % which was significantly greater than that of monthly SP or daily trimethoprim-sulfamethoxazole (TS) [13], but lower than previously reported studies in older children and adults. The reasons for this lower efficacy are not clear, but may include inadequate drug exposure due to pharmacokinetic (PK) distinctions in children, low adherence, and/or lower immunity in this younger age range. To investigate the magnitude of drug exposure in young children and explore the impact of drug exposure on the protective efficacy of monthly DHA/PQ, retrospective PK analysis was performed using banked plasma samples from this randomized clinical trial and associations with the incidence of malaria were evaluated.

## Methods

### Ethics, consents, and permissions

Informed consent was obtained from the parent or guardian of all study participants. Ethical approval was obtained from the Uganda National Council of Science and Technology, the Makerere University School of Medicine Research and Ethics Committee, and the University of California, San Francisco, Committee on Human Research.

### Study site and participants

A randomized, controlled, open-label trial was conducted in the Tororo District of eastern Uganda, an area with intense year round malaria transmission, between June 2010 and September 2013. The clinical trial compared the efficacy and safety of three regimens [monthly SP, daily trimethoprim-sulfamethoxazole (TS), and monthly DHA/PQ] versus no therapy for the prevention of malaria in young children, as previously reported [13]. Briefly, 400 infants were enrolled and 393 randomized at 6 months of age to one of the above treatment arms. Chemoprevention was given at home without supervision from 6 months through 24 months of age. The sub-study described in this report includes only infants randomized to DHA/PQ (n = 87) and no chemoprevention (n = 89) who reached 24 months of age. DHA/PQ (tablets of 40 mg of dihydroartemisinin and 320 mg of piperaquine; Duocotecxin; Holley Pharm), was administered as a standard regimen each month (once daily targeting a total dose of 6.4 and 51.2 mg/kg of dihydroartemisinin and piperaquine, respectively, given in three equally divided daily doses to the nearest one-quarter tablet [6]).

At the time of treatment allocation, parents/guardians were given a 2-month supply of DHA/PQ and a diary with dates for dosing and check-offs to indicate administration. DHA/PQ was resupplied to maintain a 2-month supply during clinic visits. Children were permitted to eat their meals at flexible times without specifying food intake at the time of study dose.

### Clinical follow-up

Clinical follow-up procedures have been previously described [13]. Briefly, children were seen in the clinic monthly where they underwent routine evaluations including thick blood smears, assessment of use of insecticide-treated bednets, and adherence to study drugs. Caregivers were instructed to visit the clinic any time their child presented with illness. Children who presented with a fever (tympanic temperature ≥38.0 °C) or history of fever in the previous 24 h had blood obtained by finger prick for a thick smear. If the thick smear was positive for malaria parasites, the patient was diagnosed with malaria regardless of parasite density. Episodes of uncomplicated malaria were treated with artemether-lumefantrine, the current first-line treatment in Uganda, while episodes of complicated malaria or repeat episodes within 14 days of a prior treatment were treated with quinine. Following treatment for clinical illness, children continued to receive DHA-PQ for chemoprevention at their regularly scheduled times, and were not excluded from further analysis.

### Pharmacokinetic sample collection and PQ concentration measurements

All children assigned DHA/PQ provided blood samples for PQ quantitation. Samples were collected by phlebotomy into EDTA tubes either during a regularly scheduled clinic visit performed every 4 months or at the time a child presented to the study clinic with clinical malaria. All samples were centrifuged within 60 min at 2000×*g* for 10 min; plasma was harvested and transferred to cryovials and stored at −80 °C. Samples were later shipped to the Drug Research Unit of the Department of Clinical Pharmacy at the University of California San Francisco, for determination PQ levels which were analysed using liquid chromatography and tandem mass spectrometry, using a deuterated PQ (PQ-d_6_) as an internal standard, as previously described [14]. The lower limit of quantification (LLOQ) was 1.5 ng/ml and inter- and intra-day variability was <15 %.

### Population pharmacokinetic analysis

PQ concentration measurements were analysed using a previously published population pharmacokinetic (PK) model generated through a clinical trial investigating DHA/PQ as treatment for children aged 2–10 years from Burkina Faso [15]. Nonlinear mixed effects modelling [NONMEM^®^ (version 7.3, Globomaxx LLC, Hanover, MD, USA)] was used to develop the PK model which is best described by two-transit-compartments in the absorption phase and by a three-compartment model in the distribution phase. Briefly, the selected model indicated the following parameters: model estimated oral clearance (CL/F) = 7.50 l/h (95 % CI 7.14–7.88), median value of apparent total volume of distribution (VD/F) = 3850 l (range 1710–7270), median value of half-life (t_1/2_) = 23.2 days (range 14.8–31.3) and median value of the area under the plasma concentration–time curve from time point 0 to day 45 (AUC_day0–45_) = 36.4 h µg/ml (range 9.61–93.0). Since the selected model was based on capillary plasma concentrations, venous plasma concentrations were converted using a correlation described in the modelling paper [15]: ln(C_capillary_) = 0.394 × ln(C_venous_) + 2.702.

Weight was previously shown to be a significant covariate for CL/F and VD/F. Therefore, the same covariate approach was used in this current analysis in order to adjust for distinctions in the weight ranges between the two studies. Weights for the current study ranged from 6 to 12 kg at the time of PQ sample collection; thus all samples were divided in 7 weight strata for 6, 7, 8, 9, 10, 11 and 12 kg. All clearance and volume parameters were allometrically scaled to the mass of the weight strata divided by the median value of the population and then raised to a power of 0.75 (for clearance) or 1.0 (for volume) [15]. In this analysis, the median weight value of the studied population was obtained by first taking the average of the measured weights per child and then the median of these average weights. Since weights of children varied during the intervention, visual predictive checks (VPC) with predicted confidence intervals (2.5–97.5 %) were simulated for each weight strata during the last monthly dosing intervals of the intervention. A representative VPC for children in the 9 kg weight strata is shown in Additional file 1.

### Statistical analysis

#### PQ exposure scoring system

**Fig. 1:**
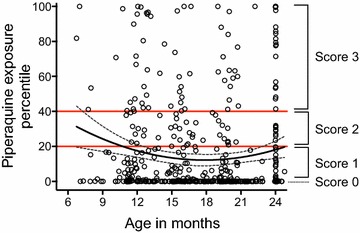
Assigned percentile per collected PK measurement and piperaquine exposure scores. Percentiles for each collected plasma sample of piperaquine, plotted against the age of the child at the time of the measurement. The *red text* displays the percentile boundaries of the piperaquine scores 0, 1, 2 and 3. *Solid line* represents best fit relationship between days since start of intervention and piperaquine exposure percentile with *dotted line* showing 95 % confidence intervals

Based on the population model, PK profiles of PQ were simulated for each of the 7 weight strata. In total, 1000 simulations were run using NONMEM^®^ for each weight strata, based on assigned DHA/PQ dosing and PK parameters previously generated in children from Burkina Faso [15]. Samples below LLOQ (1.5 ng/ml) were allocated half the value of LLOQ, 0.75 ng/ml. For each observed PQ concentration, a predicted distribution of the observations (at the specific time point when the sample was measured) was created. All observed PQ concentrations were plotted in their respective predicted distribution and assigned a probabilistic value, a percentile, describing how far away each PQ level was from the mean of the predicted distributions of observed values (Fig. 1). All percentiles were generated through statistical computing in the integrated developing environmental programme RStudio (version 0.98.1062) for the programming software R (version 3.1.2).

A PQ exposure scoring system was created based on the model-generated percentiles for each PQ concentration measurement. Based on the percentile distributions of each PQ concentration measurement, intervals were generated and assigned a PQ exposure score. A percentile of 0 was assigned a score of 0; percentiles from 0 to ≤20 a score of 1; percentiles >20 to ≤40 a score of 2; and percentiles >40 a score of 3 (Fig. 1). The average PQ exposure score per child was calculated by dividing the sum of a child’s PQ scores by the child’s number of PK measurements that were collected during the intervention. Children were then divided into three strata based on their average PQ exposure score [0 to <1 (“low”), 1 to <2 (“medium”), and >2 (“high”)].

#### Predictors of PQ exposure and impact on clinical outcomes

**Fig. 2:**
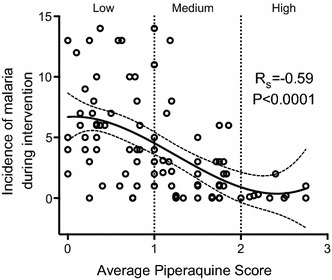
Average piperaquine score and the incidence of malaria. The average incidence of malaria per child plotted against the average adherence score. *Solid line* best fit line of generated data with *dotted line* showing 95 % confidence intervals. *R*_*S*_ Spearman’s rank correlation

All statistical analyses were performed using Prism 6.0 (GraphPad), R Version 3.1.2, and STATA version 12 (StataCorp). Descriptive statistics of the study cohort included factors associated with malaria risk in this setting [16], including housing structure type, education level of the primary care giver, maternal age, and location of residence. Comparisons between groups were made using the Chi squared test (Table 1). Multivariate linear regression was used to estimate associations between predictors of interest and average PQ exposure scores in each participant (Table 2). The non-parametric Spearman correlation was used to assess the relationship between average PQ exposure score as a continuous variable and the incidence of malaria among study participants randomized to DHA/PQ (Fig. 2).

**Table 1:** Descriptive statistics of study cohort followed from 6–24 months of age

Characteristic	No chemoprevention	Monthly DHA/PQ
Number of children followed	89	87
Female gender, n (%)	41 (46)	46 (54)
Location of residence, n (%)		
Rural, n (%)	85 (95.5)	79 (90.8)
Town, n (%)	2 (2.3)	6 (6.9)
Unknown, n (%)	2 (2.3)	2 (2.3)
Maternal age in years		
16 to <22, n (%)	30 (33.7)	25 (28.7)
22 to <36, n (%)	53 (59.6)	53 (60.9)
36 to <45, n (%)	6 (6.7)	9 (10.3)
House construction		
Non-ideal home	81 (91.0)	75 (86.2)
Ideal home	6 (6.7)	10 (11.5)
Unknown	2 (2.3)	2 (2.3)
Primary caregiver education		
Primary school or less, n (%)	69 (77.5)	74 (85.1)
More than primary school, n (%)	18 (20.2)	11 (62.5)
Unknown, n (%)	2 (2.3)	2 (2.3)

*DHA/PQ* dihydroartemisinin-piperaquine

**Table 2:** Predictors of piperaquine (PQ) exposure

	N	Mean adherence score (SD)	Unadjusted P value	Adjusted^a^ P value
Gender				
Male	40	1.02 (0.64)	Ref	Ref
Female	47	1.11 (0.76)	0.55	0.57
Location of residence^a^				
Rural	79	1.04 (0.70)	Ref	Ref
Town	6	1.13 (0.97)	0.76	0.98
Maternal age in years				
16 to <22	25	1.02 (0.67)	Ref	Ref
22 to <36	53	1.02 (0.72)	0.99	0.98
36 to <45	9	1.46 (0.66)	0.11	0.09
House construction^b^				
Non-ideal home	75	1.01 (0.71)	Ref	Ref
Ideal home	10	1.33 (0.65)	0.18	0.23
Primary caregiver education^b^				
Primary school or less	74	1.03 (0.71)	Ref	Ref
More than primary school	11	1.11 (0.72)	0.73	0.89

^a^All covariates included in multivariate model

^b^2 children with unknown location of residence, housing construction, and primary caregiver education

The protective efficacy of DHA/PQ chemoprevention was assessed using a negative binomial regression model, stratifying children randomized to DHA/PQ by their average PQ exposure score (0 to <1, 1 to <2, and ≥2, Table 3). The observation period began on the day of randomization and ended once the child reached two years of age. Incident episodes of malaria were defined as all febrile episodes accompanied by any parasitemia requiring treatment, but not preceded by another treatment in the prior 14 days. Time at risk was from the day following the initiation of study drugs to the last day of observation, minus 14 days after each treatment for malaria. The incidence of malaria was calculated as the number of episodes per person years at risk (ppyr). Measures of association were expressed as protective efficacy (PE = 1 minus the incident rate ratio) during the intervention. Multivariate models adjusted for factors associated with malaria risk in this setting, as above. Two-sided p values were calculated for all test statistics and *P* < 0.05 was considered significant.

**Table 3:** Protective efficacy of DHA/PQ when stratified by PQ exposure

Treatment arm	PQ strata^a^	N	Number of cases	PYAR	Incidence of malaria	Univariate		Multivariate^b^	
						PE (95 % CI)	*p* *value*	PE (95 % CI)	*p* *value*
No chemoprevention	–	89	723	105.9	6.83	–	–	–	–
	Low	40	242	50.7	4.77	31 % (5–50 %)	0.023	31 % (6–49 %)	0.017
Monthly DHA/PQ	Medium	37	115	50.8	2.26	67 % (53–77 %)	<0.001	67 % (54–76 %)	<0.001
	High	10	3	15.0	0.20	97 % (91–99 %)	<0.001	97 % (89–99 %)	<0.001

*DHA/PQ* dihydroartemisinin/piperaquine, *PYAR* person years at risk, *PE* protective efficacy

^a^PQ strata based on average PQ exposure score [0 to <1 (“low”), 1 to <2 (“medium”), and >2 (“high”)]

^b^Multivariate model includes housing location, type, maternal age, and education level of primary care giver

## Results

### Study cohort and clinical outcomes

Children were enrolled into a previously described, open-label clinical trial of anti-malarial chemoprevention [13] where the assigned treatment doses were not directly observed and administered at home. In this report, children randomized to either no chemoprevention or DHA/PQ and who completed the chemoprevention intervention at 24 months of age were analysed. Of 200 children initially enrolled, 89 children randomized to no chemoprevention and 87 children randomized to DHA/PQ were followed to 24 months of age. Children randomized to DHA/PQ had PQ measurements performed serially and at variable times during the intervention. Baseline characteristics independently associated with malaria risk in this setting, including location of residence, maternal age, housing construction type, and primary caregiver education [16], were similar between the two groups (Table 1). The incidence of malaria in the 89 children randomized to no chemoprevention and followed to 24 months of age was 6.83 episodes per person year at risk (ppyr), and was 3.09 episodes ppyr among children randomized to DHA/PQ (PE 54 %, 95 % CI 38–66 %, P < 0.001). The proportion of assigned DHA/PQ doses reportedly administered at home based on diaries completed by primary caregivers was 99.2 %.

### PQ exposure measurements and pharmacokinetic modelling

As DHA/PQ was provided at home by a caregiver without direct observation in the clinic, plasma PQ levels were measured in banked plasma samples to better estimate PQ exposure. Precise information as to date of sample collection and the date of when the prior dose was scheduled to be administered was available. An average of 5.4 samples were collected per child during the period of DHA/PQ administration (493 samples total). Of these, 200 (41 %) samples were collected at times when children presented with clinical malaria and 293 (59 %) were collected at variable times post assigned DHA/PQ dose during regular routine visits to the clinics. All samples were included in the analysis except eight plasma measurements for which there was clear evidence that samples were collected immediately after participants took DHA/PQ off schedule in response to symptoms of malaria.

Pharmacokinetic models were used to estimate PQ exposure incorporating time since last DHA/PQ dosing and weight. For each observed PQ concentration, a predicted percentile was assigned, and a PQ exposure scoring system was created based on this model. Each child was assigned an average PQ exposure score and children were further stratified into three groups based on their average PQ exposure score as described above. 40 children received an average PQ exposure of 0 to <1 (“low”), 37 children received an average score of 1 to <2 (“medium”) and 10 received an average score of >2 (“high”) (Fig. 1).

### Predictors of PQ exposure

As factors associated with malaria risk may also be associated with adherence to the treatment intervention and/or drug metabolism, associations between baseline characteristics and PQ exposure scores were explored. These included gender and markers of socioeconomic status, including age and education level of the primary caregiver, housing location, and type of housing [16]. In both univariate and multivariate analysis, none of these indicators were significantly associated with PQ exposure (Table 2).

### PQ exposure strongly associated with incidence of malaria

Given the variability in PQ scores among children randomized to DHA/PQ, the relationship between PQ exposure and the incidence of malaria among these children was determined. Average PQ exposure as a continous measure was strongly associated with malaria incidence (Fig. 2). Children with the highest average PQ scores had significantly less malaria than children with the lowest exposure scores (R_s_ −0.59, P < 0.001).

The protective efficacy of monthly DHA/PQ was then estimated for children stratified into three categories based on average PQ exposure scores. There was a clear dose relationship in protective efficacy with increasing PQ exposure. In univariate analysis, children in the “low” PQ exposure strata had 31 % less malaria (95 % CI 5–50 %); children in the “medium” exposure strata had 67 % less malaria (95 % CI 53–77 %); and children in the “high” exposure strata had 97 % less malaria (95 % CI 89–99 %) when compared to children given no chemoprevention (Table 3). In multivariate analysis controlling for factors associated with malaria risk in this setting, the results were nearly identical (Table 3). Together, these findings suggest that the protective efficacy of DHA/PQ chemoprevention in young children is directly related to adequate drug exposure, and that in children with the highest drug exposure, monthly DHA/PQ is nearly 100 % protective against malaria.

## Discussion

In this study, the results of a previously published clinical trial of monthly DHA/PQ for the prevention of malaria in young children were re-analysed, after assessment of PQ exposure through serial venous plasma PQ measurements in 87 children randomized to DHA/PQ. Despite nearly 100 % reported adherence, a wide variability of PQ exposure was observed with the majority of values less than expected based on a previous model with 89 % of children having model-generated percentiles averaging ≤40 with the remaining 11 % of children having percentiles averaging >40. This variability could not be explained by gender and/or socioeconomic factors associated with malaria risk in this setting. In multivariate models, increasing PQ exposure was associated with improved efficacy of DHA/PQ, and in children with the highest drug exposure, DHA/PQ chemoprevention was nearly 100 % protective against malaria.

DHA/PQ is increasingly being considered for IPT in several settings. When given as directly observed therapy, monthly DHA/PQ offered protective efficacy of 98 % against malaria in Thai adults [11]. Similarly, when given as directly observed IPT to Ugandan school children 6–14 years of age, monthly DHA/PQ had a protective efficacy of 96 % in preventing malaria. With PK modelling, the protective efficacy of DHA/PQ in Thai adults was found to be PQ concentration dependent, with plasma PQ trough concentrations of 6.7 and 20 ng/ml reducing the hazard of malaria by 50 and 95 %, respectively [17], while during the trial no malaria occured in participants with a trough concentration above 31 ng/ml [11], underscoring the importance of consistent PQ exposure.

In this study, significant intersubject variability was observed in PQ exposure using serial concentration measurements made over 2 years of longitudinal follow-up. There are several potential reasons for this variability. One possibility is inadequate adherence with at home study drug administration. Although self-reported adherence was nearly 100 %, it is possible that recall by caregivers was an inadequate measure of adherence. This is supported by intrasubject variability in exposure estimates seen over time for some children (data not shown) that would suggest inconsistent study drug administration. Another possibility is that these young children were inadequately dosed with DHA/PQ, especially at lower weights. Through PK modelling from Thai adults, pharmacokinetic simulations for paediatric populations suggest that the incidence of malaria infections over 1 year could be reduced by 70 % for children weighing 8–12 kg if they were administered higher doses than currently recommended by the manufacturer [17]. The vast majority of children in the present study had model generated percentiles of ≤40 utilizing an earlier model stemming from DHA/PQ use in older children aged 2–10 years, suggesting that the youngest children (<2 years), as enrolled in this study, may be inadequately dosed due to pharmacokinetic distinctions for children of different age groups.

Finally, PQ resistance has been reported to increase the in vitro IC_50_ significantly when resistance was induced in vitro [18]. In a recently published study using isolates obtained from this clinical trial, ex vivo drug sensitivity decreased for PQ over time [19], although there was not clinically relevant evidence for PQ drug resistance in this setting, as has been previously reported in other parts of Uganda [20]. It is possible that higher PQ exposure is needed to offset these changes to provide the best protection against malaria.

There are some limitations of this study. Since study drug administration was not directly observed, it is not possible to definitively distinguish between adherence versus PK distinctions in very young children as the cause for the variability of PQ drug exposure. Future studies of PQ pharmacokinetics and the relationship between PK exposure and clinical outcomes are now underway in very young children receiving directly observed therapy. Likewise, although an average of five venous plasma measurements in children randomized to DHA/PQ were obtained during this study, sampling was variably timed following the scheduled day of study drug administration which contributed to the variability. Lastly, this analysis was dependent on a model generated in older children with capillary plasma measurements and measurements were from venous plasma samples. Although a published correlation equation for older children [15] was used to correct for this difference in matrix, the correlation was based on samples collected immediately post-dosing which may not be applicable to samples collected many days post-dosing as done in our study.

## Conclusions

In conclusion, the protective efficacy of DHA/PQ chemoprevention in young children is directly related to the extent of exposure to long acting PQ, and in children with the highest PQ exposure, DHA/PQ chemoprevention is nearly 100 % protective against malaria. Strategies to increase PQ concentrations in young children, including directly observed therapy or optimized dosing guidelines for this distinct patient population, should be utilized or developed to improve the efficacy of this promising malaria control strategy.

## Additional file

10.1186/s12936-015-0908-8 Visual predictive check of observed, capillary corrected plasma concentrations of piperaquine, measured in children from the 9 kg weight strata during the last monthly dosing interval of the intervention. Black line represents the 50th percentile, with light grey lines representing predicted confidence intervals (2.5 % - 97.5 %). Red points represent the observed, capillary corrected plasma concentration of piperaquine. In this weight strata, the majority of the observations fall below the 2.5 % limit of the confidence interval.
