# Supplementary figures and images for: Variable piperaquine exposure significantly impacts protective efficacy of monthly dihydroartemisinin-piperaquine for the prevention of malaria in Ugandan children
Source: Malar J. 2015 Sep 24;14:368. doi: 10.1186/s12936-015-0908-8 (PMC4582734; doi:10.1186/s12936-015-0908-8)

**Simulated PK profile for weight strata 9 kg**

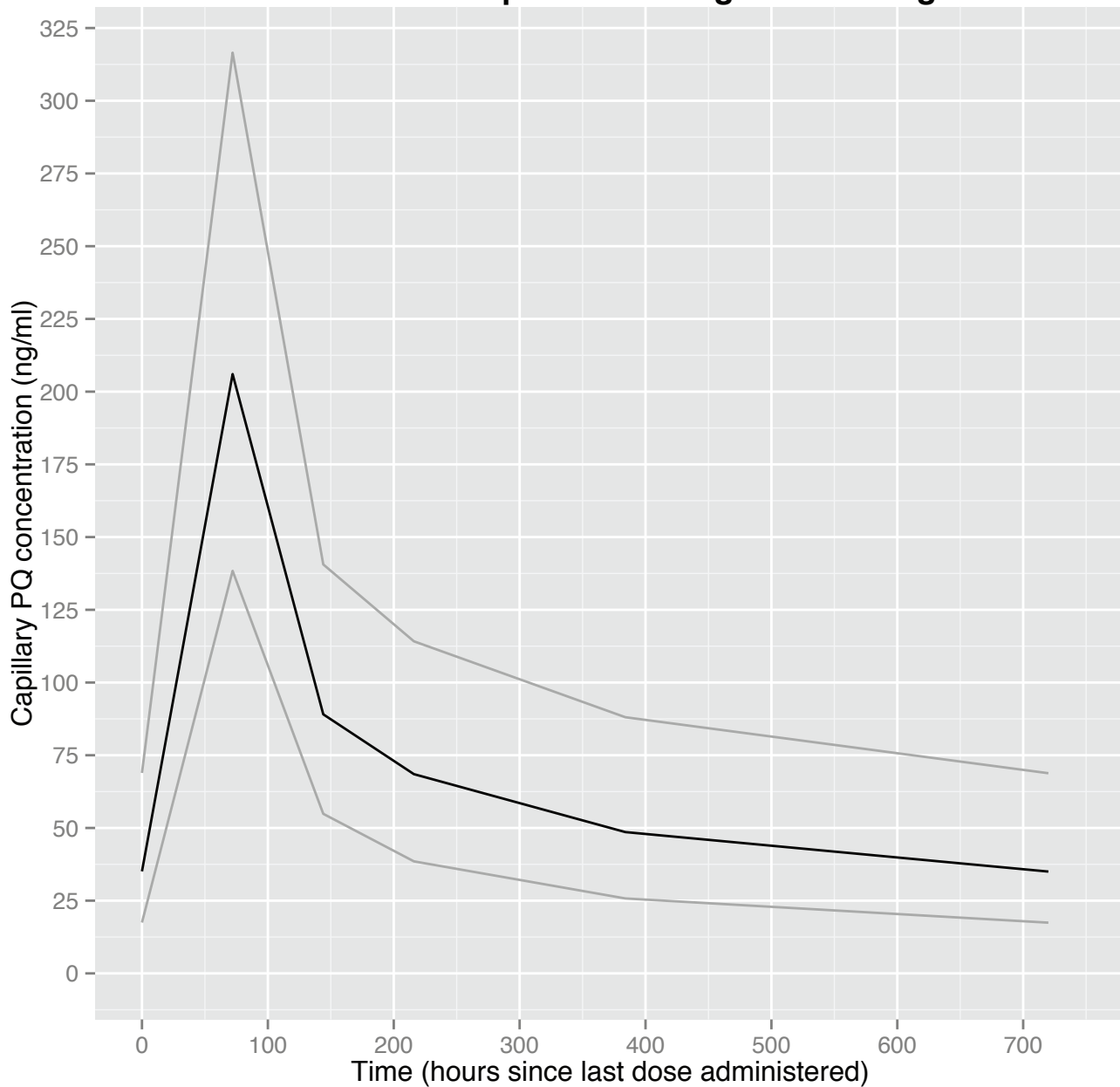

Supplement: Supplementary file 1 — 10.1186/s12936-015-0908-8 Visual predictive check of observed, capillary corrected plasma concentrations of piperaquine, measured in children from the 9 kg weight strata during the last monthly dosing interval of the intervention. Black line represents the 50th percentile, with light grey lines representing predicted confidence intervals (2.5 % - 97.5 %). Red points represent the observed, capillary corrected plasma concentration of piperaquine. In this weight strata, the majority of the observations fall below the 2.5 % limit of the confidence interval. [file 12936_2015_908_MOESM1_ESM.pdf]
